# Right ventricular (RV) velocity measurements using high resolution spiral myocardial phase velocity mapping (PVM)

**DOI:** 10.1186/1532-429X-15-S1-P133

**Published:** 2013-01-30

**Authors:** Robin Simpson, Jennifer Keegan, David N Firmin

**Affiliations:** 1 NIHR Cardiovascular Biomedical Research Unit, Royal Brompton Hospital, London, UK; 2 Imperial College, London, UK

## Background

PVM is capable of accurately and reproducibly measuring myocardial velocities in the LV [1]. However, analyzing RV motion is more difficult because of the thinness of the free wall and its asymmetrical geometry. Currently, the most commonly used technique for assessing RV velocities and function is Tissue Doppler Imaging (TDI) where the high temporal resolution allows the detailed analysis of fine features of motion (small peaks in velocity during isovolumic contraction (IC) and isovolumic relaxation (IR) [2], for example). However TDI is restricted by inadequate acoustic windows and cannot comprehensively assess velocities over the entire RV. This study aims to establish that high resolution spiral PVM is capable of reproducibly measuring RV free wall velocities and that, consequently, it has a future role in assessing RV function.

## Methods

K-space is covered with 13 spiral interleaves (12 ms duration, TR 21ms). Navigator-gated reference and velocity-encoded data (25 cm/s through-plane) are acquired in consecutive cardiac cycles following a single dummy cycle. The acquired spatial resolution is 1.4 x 1.4 x 8 mm (reconstructed to 0.7 x 0.7 mm). Retrospective gating allows full coverage of the cardiac cycle with 60 phases per RR-interval (reconstructed temporal resolution 14-20 ms). Basal, mid and apical short-axis slices were acquired in 10 healthy volunteers on a Siemens Skyra 3 Tesla scanner. The mid slice was also acquired on a second day to assess inter-study reproducibility. Longitudinal velocities averaged over the RV free wall were extracted and peak velocities and times to those peak (TTP) velocities were measured and normalized to a fixed systolic (350 ms) and diastolic length (650 ms). The reproducibility of mid slice values was determined as the mean (+/- SD) of the signed differences of the two measurements made on different days.

## Results

**Figure 1:**
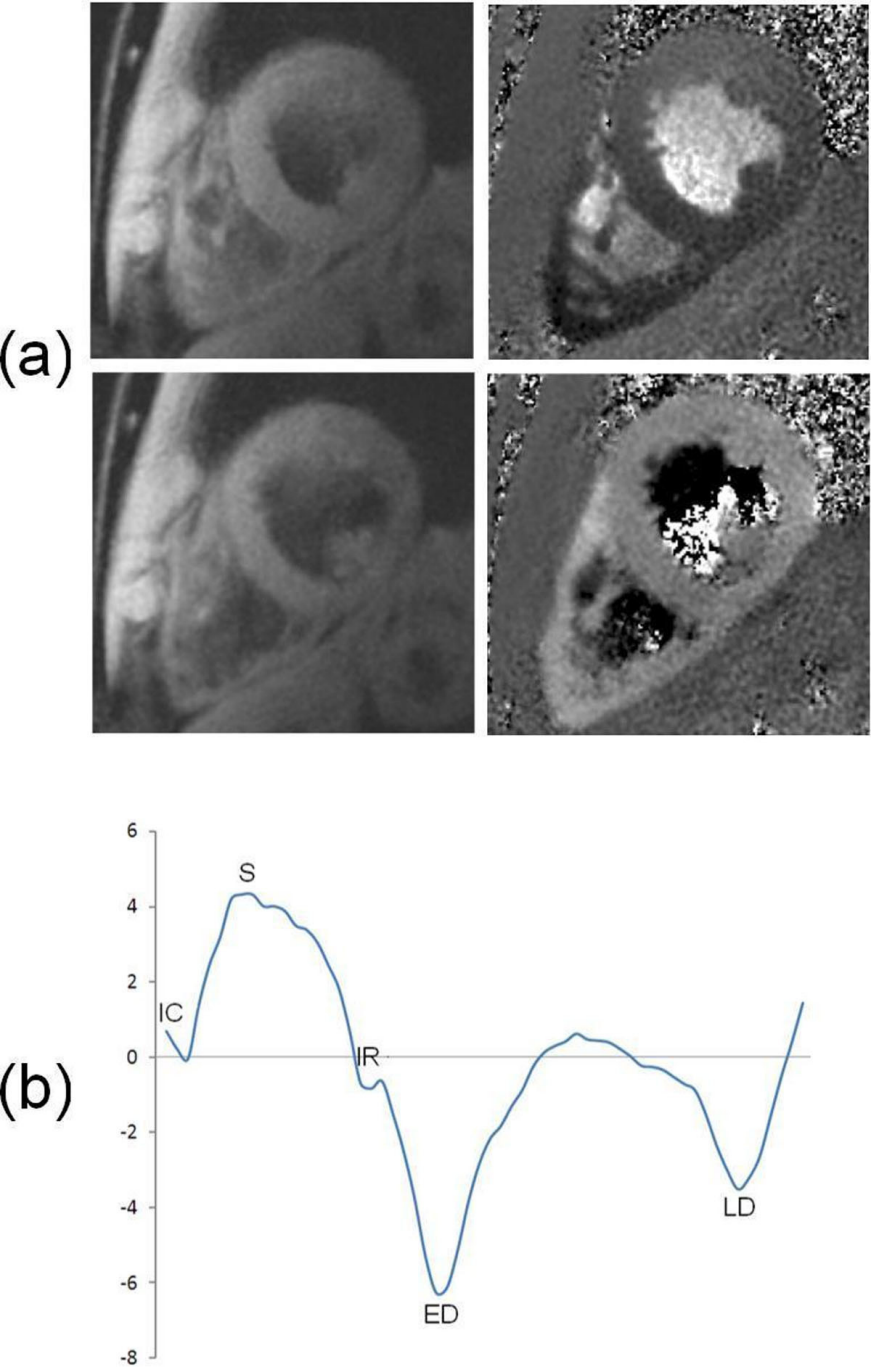
Peak velocities are at the lower end of the healthy subject range defined by previous TDI studies [2]; this is likely to be due to reduced temporal resolution and to averaging the longitudinal velocities over the entire free wall instead of in a small region of interest. (a) Example magnitude images (left) and velocity maps (right) at the time of peak systolic (S, top) and peak early diastolic (ED, bottom) velocity. (b) Example RV wall average velocity-time curve. The 5 phases of right ventricular motion (isovolumic contraction (IC), systole (S), isovolumic relaxation (IR), early diastole (ED) and late diastole (LD)) observed with TDI [2] can clearly be seen.

The high spatial resolution allowed the analysis of the thin RV free wall in all slices and in all volunteers on both occasions - example data are shown in Figure 1. Mean+/-SD velocities and TTP velocities for systolic, early diastolic and late diastolic peaks in the basal, mid and apical short axis slices are shown in Table 1, and are highly consistent between subjects. Inter-study reproducibilities of peak systolic, early diastolic and late diastolic velocities were excellent (0.21±1.31, 0.15±1.13and 0.69±2.39cm/s respectively) as were the interstudy reproducibilities of the corresponding TTPs (11.6±78.2, -13.1±30.6 and -6.67±19.9 ms respectively). The high temporal resolution of the sequence allowed detection of IC and IR (Figure 1) in 22 out of 40 velocity-time curves.

**Table 1:** Mean +/- SD peak and TTP values for the ten volunteers

Parameter	Mean+/-SD velocity for 10 volunteers (cm/s)	Mean+/-SD normalized TTP for 10 volunteers (ms)
BASE		

Peak systolic	7.29±1.65	146.6±50.3

Peak early diastolic	-7.83±2.00	495.6±22.4

Peak late diastolic	-4.61±2.10	903.1±19.5

MID		

Peak systolic	5.08±1.67	152.7±72.2

Peak early diastolic	-5.67±1.29	487.3±24.3

Peak late diastolic	-2.42±2.52	884.6±24.7

APEX		

Peak systolic	2.98±0.82	188.6±115.0

Peak early diastolic	-3.34±0.89	508.6±35.8

Peak late diastolic	-1.87±0.64	900.1±28.2

## Conclusions

PVM can be used to measure RV free wall velocities with a high degree of reproducibility. PVM is potentially a more flexible and comprehensive modality for assessing regional RV motion than TDI.

## Funding

The authors acknowledge the support of Heart Research UK, Imperial College London and NIHR Royal Brompton Cardiovascular Biomedical Research Unit.
